# Miniature, multi-dichroic instrument for measuring the concentration of multiple fluorophores

**DOI:** 10.1364/BOE.516574

**Published:** 2024-03-14

**Authors:** Konstantin Grygoryev, Huihui Lu, Simon Sørensen, Omid Talebi Varnosfaderani, Rachel Georgel, Liyao Li, Ray Burke, Stefan Andersson-Engels

**Affiliations:** 1 Tyndall National Institute, Lee Maltings Complex, Dyke Parade, Cork, Ireland; 2 Department of Physics, University College Cork, College Road, Cork, Ireland

## Abstract

Identification of tumour margins during resection of the brain is critical for improving the post-operative outcomes. Due to the highly infiltrative nature of glioblastoma multiforme (GBM) and limited intraoperative visualization of the tumour margin, incomplete surgical resection has been observed to occur in up to 80 % of GBM cases, leading to nearly universal tumour recurrence and overall poor prognosis of 14.6 months median survival. This research presents a miniaturized, SiPMT-based optical system for simultaneous measurement of powerful DRS and weak auto-fluorescence for brain tumour detection. The miniaturisation of the optical elements confined the spatial separation of eight select wavelengths into footprint measuring 1.5 × 2 × 16 mm. The small footprint enables this technology to be integrated with existing surgical guidance instruments in the operating room. It’s dynamic ability to subtract any background illumination and measure signal intensities across a broad range from pW to mWs make this design much more suitable for clinical environments as compared to spectrometer-based systems with limited dynamic ranges and high integration times. Measurements using optical tissue phantoms containing mixed fluorophores demonstrate correlation coefficients between the fitted response and actual concentration using PLS regression being 0.95, 0.87 and 0.97 for NADH, FAD and PpIX , respectively. These promising results indicate that our proposed miniaturized instrument could serve as an effective alternative in operating rooms, assisting surgeons in identifying brain tumours to achieving positive surgical outcomes for patients.

## 1. Introduction

Bio-marker based tissue discrimination has a particular advantage in diagnostics and surgical guidance because it allows quantitative rather than subjective identification. This is of particular importance in brain surgery and tumour resections where the best clinical outcome is directly dependant on the completeness of tumour removal while retaining maximum healthy brain tissue [1]. To date, the bulk and marginal brain tumour discrimination has been achieved using an exogenous contrast agent, 5-ALA [2]. This agent is accumulated in tumour tissues due to disrupted blood-brain barrier and metabolised into fluorescent PpIX by the tumour cells. The use of 5-ALA has been shown to significantly improve the accuracy of tumour resection and increase the patient survival rates [3]. Despite these improvements, definitive delineation between tumour infiltration zone and healthy brain tissue still remains challenging, primarily because the surgeon must make subjective decisions regarding resection margins based on perceived strengths of fluorescence [4–6].

To address this issue, many clinical studies have examined spectroscope-based systems designed to detect or quantify optical differences between tumour and healthy brain [5,7–12]. Such instruments often utilize a fiber-optic probe and several light sources to measure tissue diffuse reflectance and the intensity of fluorescence induced by PpIX [5,12] or endogenous fluorophores [7,9] and build an excitation-emission matrix (EEM). One potential obstacle to clinical adoption is that the spectroscope-based instruments require integration times on the order of 100 ms to detect a low-level fluorescent signal making such systems easily saturated by strong background illumination of the operating room. Instruments capable of spatially separating strong and weak intensity signals and coupling them to dedicated detectors with matching dynamic range, provide a strong alternative to the spectroscope-based setups for clinical measurements. Such system can become very compact for straight-forward adaptation to the clinical workflow [13,14]. Also, illumination and signal acquisition of such systems can be modulated in kHz frequency range. Fast illumination and sampling makes it possible to accurately account for dynamically changing ambient lighting such that exists in operating rooms due to movement of surgical staff under strong surgical lamps. A second benefit of fast illumination is decreased photo-bleaching of endogenous fluorophores or 5-ALA induced PpIX due to reduced exposure time. The combined effect is an improved signal to background ratio. The photon detection efficiency can be improved by specifying the bio-marker spectral bands at the illumination stage with light sources of required wavelengths [15]. In addition rather than a single detector, using an array of avalanche photodiodes (APD) is an effective method of detecting low level fluorescent signals [16]. Finally, such instrument architecture allows for implementation of calibration algorithms that account for distorting factors such as tissue optical properties, auto-fluorescence and fluorescent photoproducts to derive bio-marker concentration [12,17–25].

There are several challenges remaining with quantitative measurements of auto-fluorescence. First, the difference in signal strengths between diffuse reflectance and auto-fluorescence is relatively large. As such, the dynamic range of the whole instrument can be a limiting factor to quantification. Second, the molecular composition of tissues is complex. To discriminate between tissue types more accurately requires measuring concentrations of more than one bio-marker that have, although different, but overlapping fluorescence profiles. Third, clinical adoption of any new technology requires minimal disruption to surgical workflow while providing significant improvements to surgical outcomes. Hence, this study presents a miniaturized, multi-spectral, silicon photo-multiplier (SiPMT) optical instrument, capable of continuously compensating for ambient illumination while performing contact point measurements of high intensity diffuse reflectance (DRS) and weak fluorescence signals of NAD(P)H and FAD. The instrument presented builds on our previous studies [26,27]. The current instrument extends the dynamic range for DRS measurement using different source detector fibre combination coupled to an additional photodiode. The bio-marker specificity and crosstalk were improved by use of laser source illumination, rather that LEDs. The detection module used, consisted of eight SiPMT mounted on an PCB with embedded ADC with significantly better signal to noise ratio as compared with previous prototypes. Furthermore, the small footprint of SiPMT allowed integration of miniaturized multi-spectral optics designed for spatial separation of wavelengths. This arrangement significantly reduced the instrument dimensions, removing air gaps and shortening the light path. These technical improvements enabled this instrument to discriminate between three fluorophores with varying concentrations, mixed in an optical brain tissue phantom. As more accurate tissue discrimination can be achieved my measuring concentrations of multiple fluorescent bio-markers [28,29], this capability expands the range of surgical guidance applications and makes such system more suitable for clinical translation. In this regard, the reduced footprint allows for easier integration with current, surgical guidance instruments. Such form-factor and functionality has direct application to improving the outcomes of brain tumour resections through integration into existing surgical navigation or imaging systems and providing quantitative information to the surgeon about the tissue pathology.

## 2. Materials and methods

### 2.1 Instrumentation

**Fig. 1.**
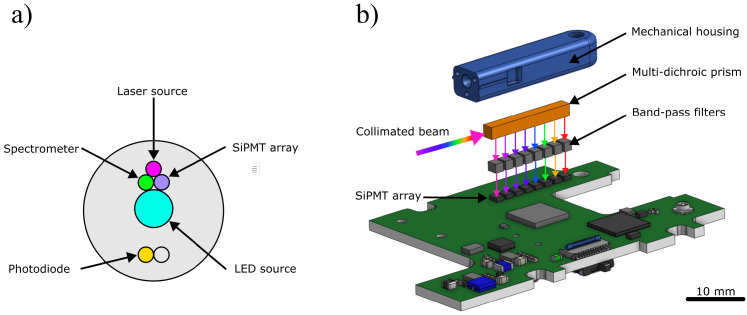
a) The schematic of fibre-optic probe end, showing the relative positions of fibres connected with respective light sources and detector instruments. b) Arrangement of miniaturized optics on PCB with SiPMTs.

The sample illumination, DRS and AF signal collection was achieved using a custom multi-fibre probe (ART Photonics, Germany). The probe consisted of six separate fibres as shown in Fig. 1(a). The illumination was achieved through two fibres. One fibre delivered light from five individual laser sources (core ∅ 200 µm, NA = 0.2), the second fibre delivered light from three LEDs (core ∅ 600 µm, NA = 0.39). Two out of three collection fibres (core ∅ 200 µm, NA = 0.2) patched directly to the light to custom SiPMT array and photodiode (DET10A2, ThorLabs, UK). A third collection fibre was coupled to a spectrometer (NIR-VIS, Wasatch, USA) through an ND filter with value of 0.5. The ND filter was used to reduce the effects of spectrometer saturation by DRS signal. The spectrometer integration time was set to 8 ms. The DRS collection fibre was positioned at a distance of 1 mm from the centre of LED illumination fibre. This arrangement was used to extend the upper limit of the dynamic range of the entire system to 22 mW. A single 200 µm fibre (colored white in Fig. 1(a)) was used as a spare channel. The multi-source illumination unit was described in detail in a previous publication [27]. Briefly, the unit consisted of 300, 340, 390, 440, 470, 515, 590 and 680nm individual sources, synchronised using an multiplexed analog output module (cDAQ 9174, National Instruments, USA). As mentioned above, the 300, 340 and 590 nm wavelengths were LEDs and coupled into single 600 µm fibre of the probe. The remaining wavelengths were generated using laser diodes, all actively aligned and coupled into single 200 µm fibre of the probe.

As shown in Fig. 1(b), the collected auto-fluorescence was spectrally divided into eight channels using a custom fabricated miniature (1.5 x 2 x 16 mm), multi-dichroic prism (Laser 2000, UK). The prism was constructed from eight dichroic elements, with cut-off wavelengths matching those of the illumination source referred to above. The dimensions and spacing of each element allowed the prism to be positioned over an array of SiPMTs (Onsemi MicroFC-10010-SMT-TR1, Farnell, Ireland). Furthermore, the light emanating from each channel of the prism was patched through an array of spectrally-matched optical bandpass filters measuring (1.5 x 1.5 x 1 mm) to attenuate cross-talk (Laser 2000, UK). The auto fluorescence signal was then amplified and digitised using a circuit and a micro-controller embedded on the PCB. The analog signal amplification circuit was described in detail by our previous publication [30]. The analog to digital conversion (DAC) was achieved by integrating a micro-controller (STM32WB55CGU6, ST Microelectronics, Switzerland), a 24-bit DAC chip (ADS1258IRTCR, Texas Instruments, USA) and other supporting electrical components with the analog amplification circuit of the SiPMTs. The sampling rate (data rate) was set to 685 Hz per channel. The electronics integration resulted in an "embedded" instrument which drastically reduced the footprint, minimised the lengths of electrical connections carrying analog voltages and critically minimised the power of electrical noise coupled into the circuit. The amplifier circuit was designed to enable the SiPMTs detect fluorescent signals in pW range. The low-light sensitivity came at the expense of ability to measure much more powerful DRS signal without saturation. As mentioned above, to address this issue, the DRS intensities for each source were measured with a separate photodiode at 10 kHz, connected to an analog-to-digital converter (NI 9202, National Instruments, USA).

### 2.2 Multi-dichroic spectra, cross-talk and SiPMT dynamic range

**Fig. 2.**
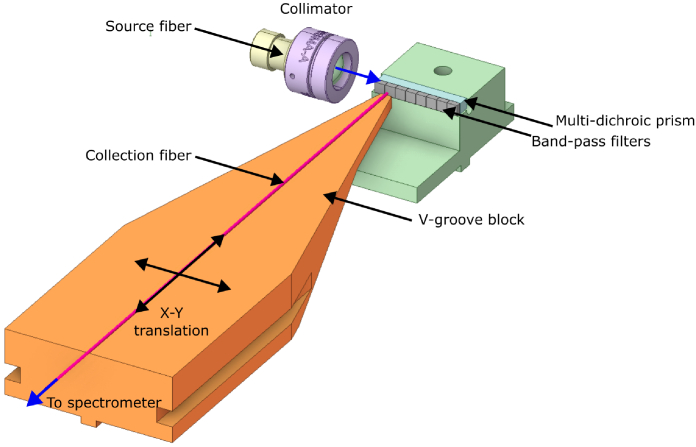
Schematic showing the fixture used for characterising transmission bands of the multi-dichroic prism and the bandpass filters.

To characterise the transmission bands of the miniaturised optics, the multi-dichroic prism and corresponding bandpass filters were coupled an optical fiber (200 µm core diameter, 0.39 NA) through a collimating lens (LA1024, ThorLabs, UK) on an 3-axis alignment stage as shown in Fig. 2. A collection fibre was attached to an X-Y translation stage and positioned in front of one channel of the miniature optical assembly. The transmission spectra through each DRS channel (source wavelengths matched to dichroic wavelengths) were measured using a high resolution spectrometer. The dynamic range of the SiPMTs and the circuit was determined by coupling light directly onto detectors, without the prism or filters, through a fibre. Using an array of calibrated neutral density filters between source and fibre, the illumination power was increased from a minimum of 2.5×10^−5^ nW up to 274.9 nW.

### 2.3 Skin and optical tissue phantoms

Invivo fluorescence was measured on skin of the pads of fingers (n=10) of a healthy volunteer. To collect the DRS values, the EEM matrix and the spectra, the optical fibre probe was placed gently in contact with a pad of each finger and then illuminated sequentially with multi-spectral source referred to above. To serve as control (zero fluorescence), an additional measurement was performed on a solid tissue phantom, here after referred to as "standard". The standard was previously made as described in [31]. Briefly, the bulk of the phantom was made using clear vulcanising silicone rubber (PlatSil, SiliGlass, Polytek, USA). The absorber (Black Silicone Pigment, MBFibreglass, UK) and scatterer consisting of silica micro-spheres (440345, Sigma-Aldrich, UK) were mixed into bulk at concentrations of 13 % and 7 %, respectively. The selected optical properties of the standard produced DRS values similar to those of the real skin at 470 and 515 nm. Following the invivo measurements, the systems performance was tested on two sets of liquid optical tissue phantoms by submerging the fibre-optic probe below the surface of the phantom to maintain measurement geometry consistent. First set of phantoms (n=20) consisted of a mixture of five FAD concentrations ( 0 µg/ml to 10 µg/ml), four absorber concentrations (0.5 % to 4 %) and constant scatterer concentration (20 %). This set was used to evaluate the effects of changing absorption values on fluorescence measurements. The second set of phantoms (n=125) contained a mixture of three fluorophores with a range of concentrations and a constant concentrations of absorber and scatterer. This phantom set was used to evaluate the system’s capability to discriminate between fluorophores with a large range and combinations of concentrations. Table 1 lists the concentration ranges of the second phantom set. This phantom set was prepared as follows. Fluorophores were added to the 20 % aqueous mixture of intralipids (Merck, Wicklow, Ireland) and India ink (Winsor and Newton black India ink, Cork, Ireland) which were used as scatterer and absorber, respectively. NADH, FAD and protoporphyrin (Merck, Wicklow, Ireland) were dissolved in each appropriate solvent as fluorophores. 4 % volume fraction of intralipids (20 %) and 0.5 % volume fraction of ink stock solution (1 %) were selected to simulate reduced scattering coefficient as 8.3 cm^−1^ at 470 nm and absorption coefficient as 0.32 cm^−1^ at 470 nm. The scattering coefficient $\mu_{s}\prime$ of intralipids and the absorption coefficient $\mu_{a}$ of Indian ink were determined using the collimated transmittance setup in our previous work [32]. The concentration of NADH, FAD and PpIX were chosen to cover a typical range of that in biological tissue. Each fluorophore was dissolved in the appropriate solvent to prepare NADH stock solution as 10 µg/ml, FAD stock solution as 1 µg/ml and PpIX 100 µg/ml. Then a series of dilutions from these stock solutions into aqueous mixture containing intralipids and ink were used to achieve the desired fluorophores concentrations (Table 1).

**Table 1.** Liquid phantom composition with five concentration of fluorophore I, five concentrations of fluorophore II, and five concentrations of fluorophores III in the aqueous mixture containing intralipids and ink

NADH (µg/ml)	FAD (µg/ml)	PpIX (µg/ml)
100	10	10
50	5	5
25	2.5	2.5
12.5	1.25	1.25
0	0	0

### 2.4 Statistical analysis

Partial least square (PLS) was used in regression analysis of three fluorophores mixed into tissue phantoms with clinically relevant optical properties. The measurement data was compiled into a matrix of n×p (125 × 36). Each row (n) corresponded to optical measurement from one phantom sample, and each column (p) corresponded to source/detector wavelength pair of the system. The SiPMT data from channels where the detection band was below the wavelength of the excitation source contained no useful information and were omitted from analysis. The mean squared errors were used to select the number of components for the PLS model. Based on this, the first four PLS components were selected to build the predication model. Leave-one-out cross validation (LOOCV) was used to evaluate the performance of the model, analysing all EEMs, except one, as the training data for the classifier, with the other spectra as the testing data and repeating this for each spectrum.

### 2.5 Data processing

The entire data set consisted of three subsets collected by three separate components described in the Instrumentation section. The photodiode collected 9 x DRS values, the SiPMT array with micro-optics collected a 9 x 8 EEM and the spectrometer collected 9 x DRS/fluorescence spectra. One of the nine measurements was performed without any illumination to record the ambient background for each system component. The other eight measurements corresponded with each wavelengths of the multi-source illumination unit. The corresponding background means were then subtracted from the measured DRS, EEM and spectra.

The measured fluorescence intensity in EEM and spectra is influenced by the optical properties of the tissue phantoms at both excitation and emission wavelengths. Furthermore, the illumination sources can have unintended fluctuations of intensity, resulting in corresponding fluctuations of fluorescence. Hence, to account for source intensity, phantom optical properties and to estimate the fluorophore concentration more accurately, the measured fluorescence was normalised to excitation and emission wavelengths intensities as described in Eq. (1):

(1) $$nFL=\frac{FL}{\sqrt{(}E_{x}\times E_{m})}$$

where nFL is normalised fluorescence intensity/counts, FL is the measured fluorescence intensity/counts, $E_{x}$ is the intensity of diffuse reflectance of excitation source and $E_{m}$ is the intensity of a second excitation source, wavelengths of witch matched that of the fluorophores. Both of these values ( $E_{x}$ and $E_{m}$ ) were essentially DRS measurements and accounted for tissue absorption of illumination and fluorescent light, respectively. For example in case of NADH, the $E_{x}$ = DRS intensity at 340 nm while $E_{m}$ = DRS intensity at 470 nm [33].

## 3. Results

### 3.1 Micro-optics and SiPMT characterization

**Fig. 3.**
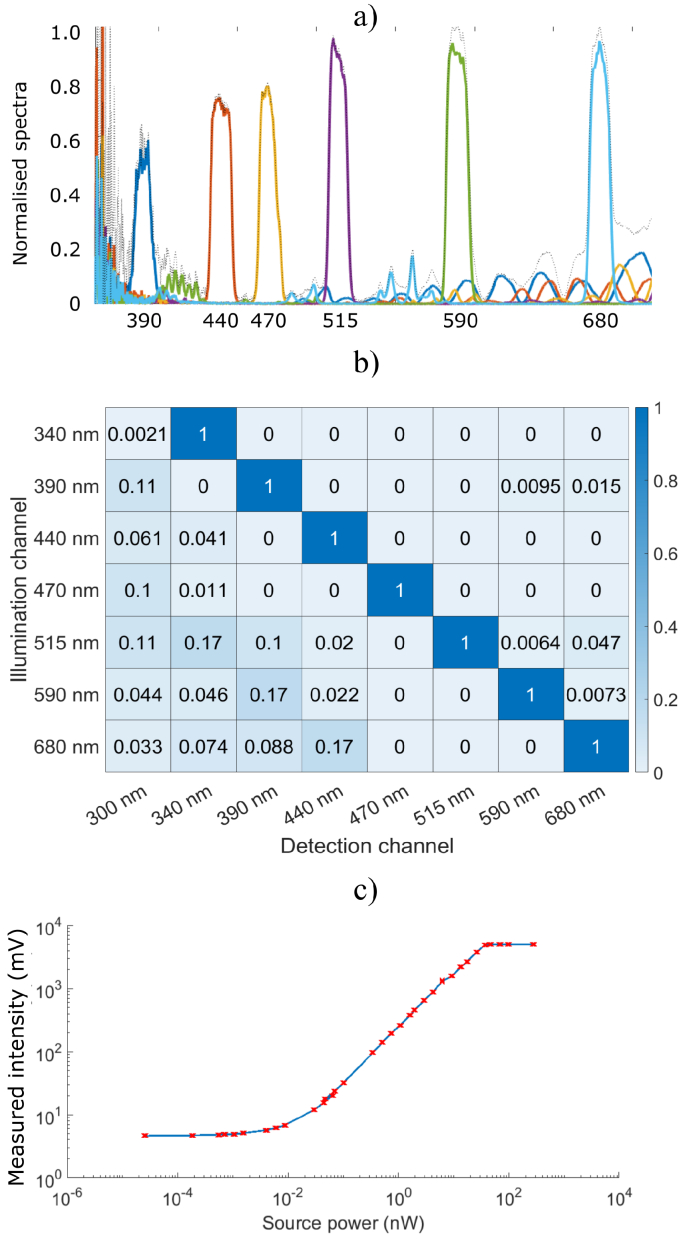
a) Normalised detection bands of the miniaturised, embedded system. b) EEM showing the percentage cross-talk between channels of the miniaturised embedded system. c) Dynamic range of the SiPMT of the embedded system.

Figure 3(a)) shows the normalised transmission bands (detection spectra) and overlap of 6/8 channels of the multi-dichroics combined with bandpass filters. The 300 nm and 340 nm channels were omitted because the spectrometer had no sensitivity below 350 nm. Figure 3(b) shows the excitation-emission matrix recorded by the SiPMT array. The data for the 300 nm excitation was omitted here because the relatively low power of the 300 nm LED, combined with low transmission properties of optics and low detector responsivity at this wavelengths resulted in no measurable DRS signal. The values above and below the diagonal, indicate the percentage of illumination light leakage through dichroic and band-pass filter surfaces into channels designated for detecting fluorescence. The crosstalk between 340 nm (NADH excitation) and 470 nm (NADH emission) was 1×10^−3^ %. The crosstalk between FAD (440-590) and PpIX (340-680) excitation and emission channels was 2.5×10^−2^ % and 2×10^−3^ %, respectively Fig. 3(c) shows the dynamic range of the SiPMT alone and associated embedded circuit. The linear response was between 0.03 nW to 37 nW or just above three orders of magnitude. The overall dynamic range of the system, including the external photodiode was 0.03 nW to 22 mW

### 3.2 Instrument bench-marking

**Fig. 4.**
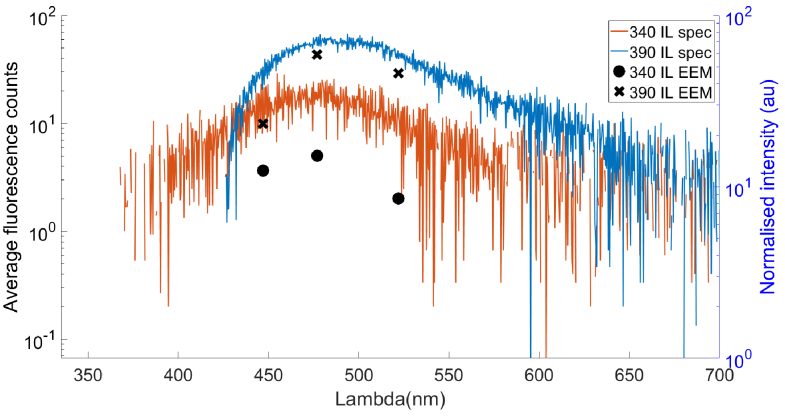
Comparison of fluorescence detection in the skin of the finger pads using the SiPMT array (crosses and circles) and a spectrometer (orange and blue traces). The fluorescence signal was measured in response to illumination by 340 nm (circles) and 390 nm (crosses) sources.

Figure 4 shows skin tissue fluorescence measured using the micro-optical instrument and a commercial spectrometer. The fluorescence intensity was measured for two excitation sources: 340 nm and 390 nm. The fluorescence peak detected by the spectrometer spanned between 400 nm and 550 nm for both excitation wavelengths. The tissue fluorescence was measured in three channels (440 , 470, 515nm) where the fluorescence intensity recorded by each channel follows a similar trend recorded by the spectrometer.

**Fig. 5.**
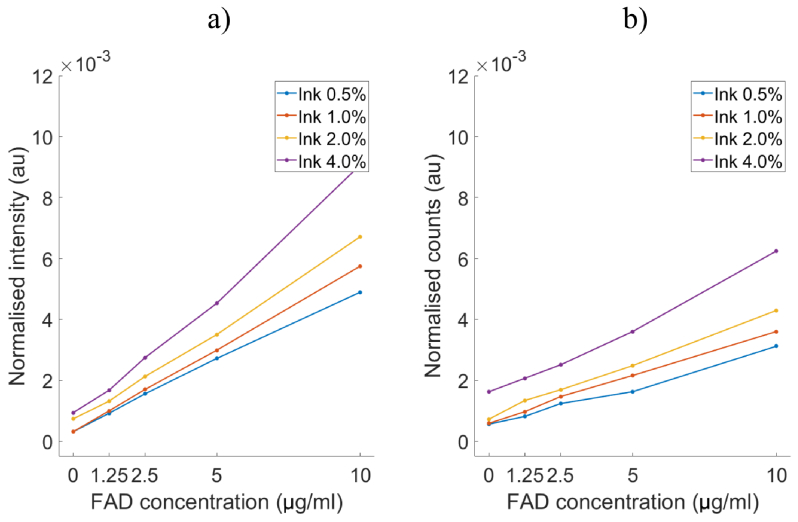
Comparison between the miniaturised embedded system and spectrometer measuring a range of FAD concentrations (0 µg/ml to 10 µg/ml) in liquid tissue phantoms with increasing concentration of absorber (0.5 % to 4 %)

Figure 5(a) and (b) show the measurements of FAD fluorescence using the embedded system and spectrometer respectively. Both measurements showed a linear correlation between fluorophore concentration and fluorescence intensity for all levels of absorber. Similar results were observed for NADH and PpIX (data not shown). Both set of measurements show a degree of dependency on the concentrating of the absorber, indicating that the normalisation method does not entirely account for source intensity or tissue phantom optical properties. For both instruments, the normalisation results in over-estimation of intensity/counts.

### 3.3 Tissue phantom fluorophore mixtures

**Fig. 6.**
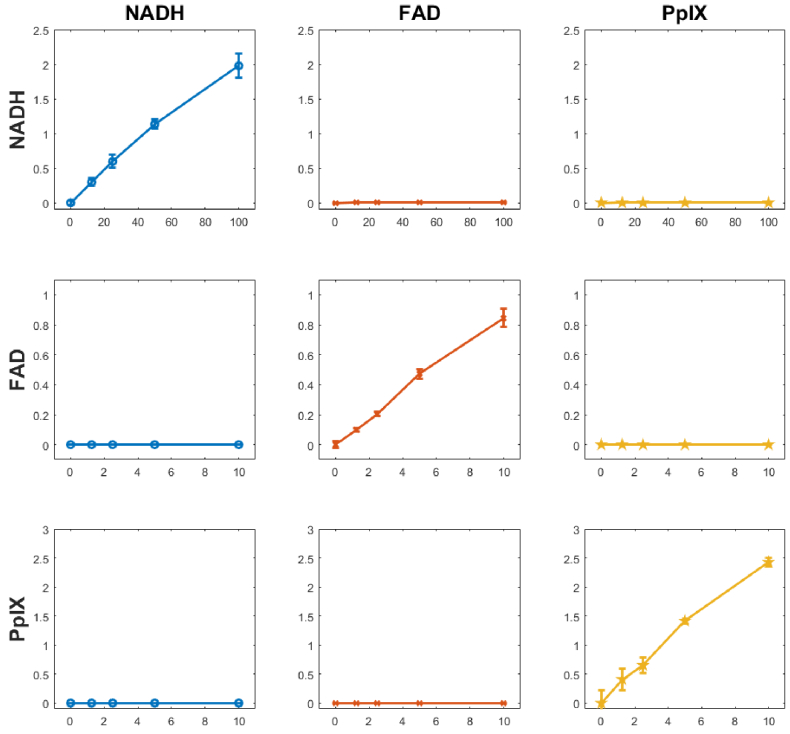
Fluorescence cross-talk indicated by measured intensities (V) of the fluorophores in response to excitation. The columns represent the fluorophore excitation channels (wavelengths), the rows represent the corresponding emission channels. X and Y axis of each sub-plot indicate fluorophore concentration in (µg/ml) and average fluorescence intensity (n=5), respectively.

**Fig. 7.**
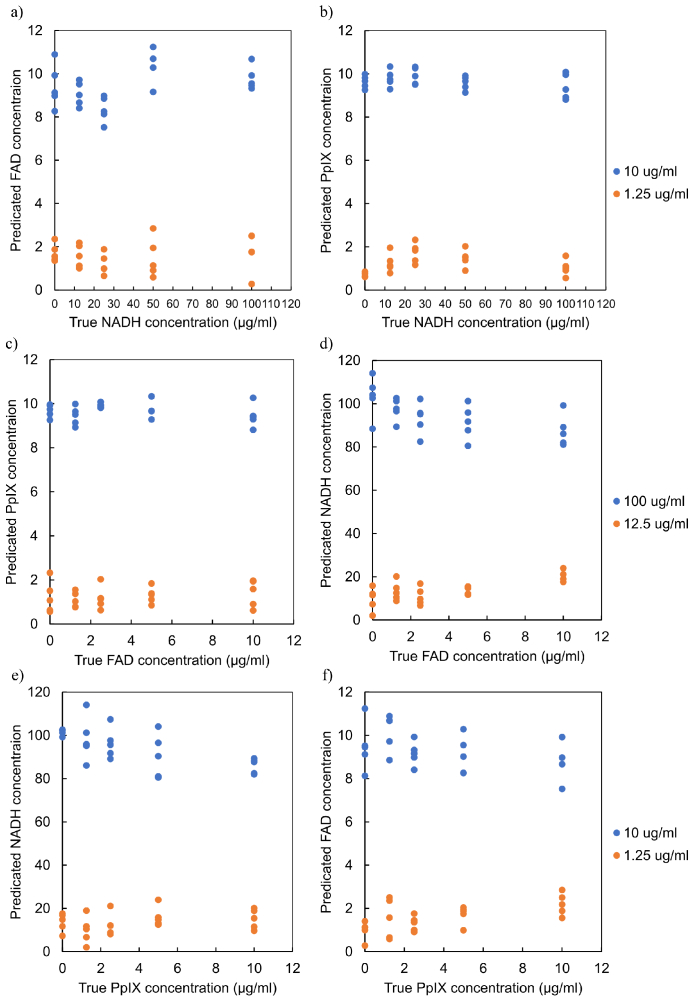
Correlation between the predicated concentration of one fluorophore and the maximum and minimum concentrations of other two fluorophores. a-b) Dependence of FAD and PpIX on NADH. respectively. b-c) Dependence of PpIX and NADH on FAD

Figure 6 shows the degree of fluorescence cross-talk for the three fluorophores and corresponding excitation wavelengths. Each fluorophore in the mixed tissue phantom showed a linear relationship with increasing concentration when the illumination source matched the absorption band. However, no significant fluorescence or concentration dependence was observed for any concentration of any fluorophore when the excitation wavelength did not match the absorption band. Figure 7 shows correlation between the predicted concentration values of one fluorophore against the true concentration of the other two. Similar to the result shown in Fig. 6, the predicted value of one fluorophore does not change with increasing concentration of the other two fluorophores.

**Fig. 8.**
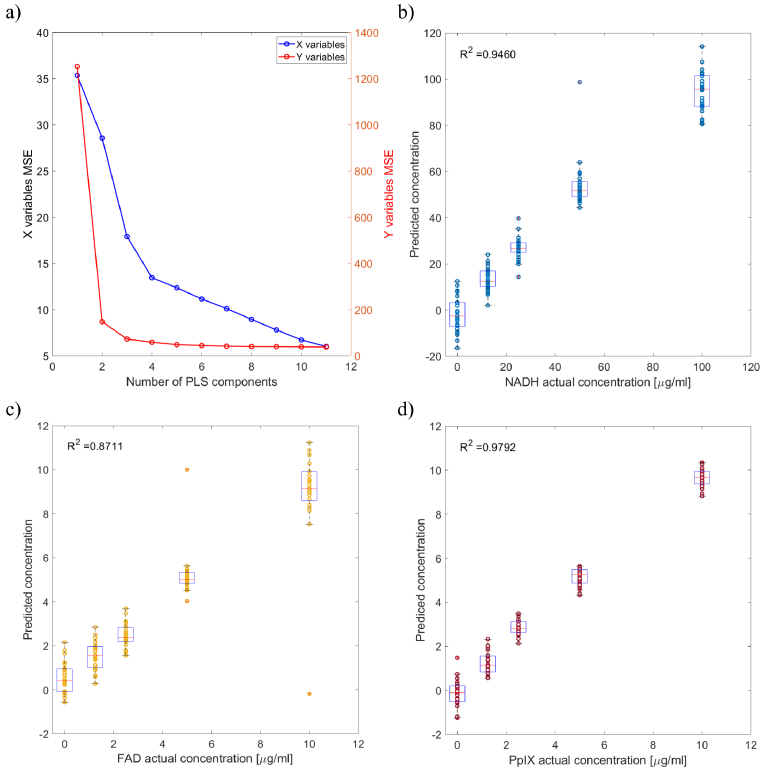
a) The relationship between mean squared error and the number of PLS components. X variables represent the excitation/emission wavelength pairs; Y variables represent different concentrations of absorbers, scatterers and fluorophores. b-d) PLS analysis and box plots of fluorescence data collected from an array of optical tissue phantoms containing NADH, FAD and PpIX fluorophores, respectively.

Figure 8(a)) shows the mean squared errors (MSE) plotted against the number of PLS components. X variables represent the excitation/emission wavelength pairs; Y variables represent different concentrations of absorbers, scatterers and fluorophores. The "elbow" of the plot was selected as the optimal number of PLS components for building the PLS regression model. It can be observed that the MSE doesn’t change significantly after the first four components. Therefore, the first four PLS components were chosen to construct the prediction model.

Figure 8(b)-c) shows the real versus predicted fluorophore concentration determined using PLS and normalised data. The PLS data also shows linear relationship between real and predicted concentrations with $R^{2}$ values of 0.95, 0.87 and 0.98 for NADH, FAD and PpIX, respectively.

## 4. Discussion

### 4.1 Optical elements and embedded system characterisation

Detection of weak auto-fluorescent signals requires effective filtering of background and excitation wavelengths where out of band attenuation is minimum OD 3. The beam-splitting surfaces of the multi-dichroic had between 90 % to 98 % efficiency according to manufacturers data. The UV channels (300 and 340 nm) were noted to have relatively low performance, likely due to material choice due to manufacturing constraints. The bandpass filters used in combination with the multi-dichroic had desired performance but only in ranges of up to 100 nm below and above the transmission band. The bandpass OD further in spectral ranges were near OD 0. The cumulative effect of the material and coatings resulted in less than optimal sensitivity of the assembly and led to an unexpected cross-talk artefact where the channels in the UV region, relative to excitation wavelengths, ’stole’ a proportion of the fluorescent signal. This artefact was shown in Fig. 3(b) where in some channels-source combinations, the total cross-talk was above 20 %. As such, sensitivity to fluorophores that have a large spectral separation between absorption and emission bands (e.g PpIX) was sub-optimal. This has a detrimental impact on detection of relatively weak fluorescent signal.

### 4.2 Dynamic range

The dynamic range of the entire system was extended by adding a separate channel for DRS measurement. The DRS collection fibre was also offset from the source fibres by over 1 mm which ensured that 1) the illumination intensity did not saturate the photodiode and 2) a larger volume of tissue phantom was sampled. The DRS data acquired using this method was used to normalise the fluorescence data and qualitatively improved the linearity. Such normalisation would not have been possible using the SiPMT array alone, as the maximum measurable power was just under 40 nW. The collected EEM matrices have all shown that four out of eight channels were saturated and recorded a maximum intensity value of ca. 5 V. The measurement of fluorescence intensity in phantoms with a range of absorption properties has shown a linear relationship with fluorophore concentration as measured by the multi-spectral system and the Wasatch spectrometer. The changing absorption values have resulted in overall change in fluorescence intensity without affecting the linear response in both instruments. The effect of absorption value indicates that the normalisation with respect to excitation or emission wavelengths is not entirely effective. The probable reason for the overestimation is the combination of the scatterer concentration, increasing absorber concentration and probe’s source-detector distance. Over a certain distance and scatterer concentration, the probability of a photon encountering an absorber is higher than encountering the detection fibre. As the result, increasing absorber concentration, results in amplified absorption of illumination wavelengths.

### 4.3 Instrument comparison

The performance of the micro-optical embedded system was compared with that of a spectrometer to ensure that collected data could be verified using an alternative instrument. Both instruments were used simultaneously on tissue phantoms with single and well as multiple fluorophores and both measured a linear response to increasing concentrations. This result shows that miniaturised, multi-spectral optical systems are a functional alternative to spectrometers. Furthermore, the spectral sensitivity of SiPMT detectors in the UV range (>300 nm) is superior to a conventional CMOS spectrometer, allowing detection of bio-marker fluorescence in the blue region of the spectra such as tryptophan (Ex 300 nm, Em 340 nm). The comparable performance of the spectrometer was achieved at the minimum integration time of 8 ms (hardware limited). An additional 8 ms were required to record background, resulting in total spectrometer measurement time of 16 ms. Despite the shortest integration time possible, all the recorded spectra that detected a fluorescence signal contained a saturated region which corresponded to the illumination wavelengths. These measurements highlight that a CMOS-based spectrometer system is not optimal for recording simultaneous DRS and weak AF signals of multiple fluorophores. It is important to point out that it is technically possible to engineer and opto-mechanical solution to address the relatively narrow dynamic range of a spectrometer. For example, adding a motorised filter wheel with a range of neutral density, long-pass and band-pass filters in front of spectrometer input can be used to select fluorophore detection bands as well as the light input power to estimate tissue attenuation of excitation and emission wavelengths. Such solution would have challenges such as added control and power modules for opto-mechanics, increased footprint and overall complexity. In addition, optical losses associated with alignment tolerance, beam collimation, condensing and coupling into another fibre or directly into a spectrometer slit are not trivial engineering challenges to solve and will present an additional barrier to future integration into clinical environment. Finally, such considerable engineering effort will result in ability to collect a high resolution spectral bands of biomarkers that have relatively broad fluorescence peaks. In such case, the high spectral resolution does not contribute additional information for fluorophore identification. The advantage of the SiPMTs with miniaturised optics is that, provided appropriate, high speed, embedded electronics are used, biomarker-specific fluorescence peaks can be selected while the measurement duration can be reduced significantly below that of a spectrometer, without the added opto and electro-mechanical complexities. The direct benefit of fast DRS and AF measurement is the ability to measure the background dynamically, either before or after illumination sequence and even between each source. Furthermore, the sensitivity of each detector can be tuned at circuit level to maximise sensitivity or to either high intensity DRS or weak AF signals.

### 4.4 System validation using fluorphore mixture

NADH fluorescence is one of the most common bio-markers of metabolic activity of the brain tissue as healthy cells convert this bio marker into the fluorescent NAD(P)H variant. Mutations in the genes associated with this metabolic process are found in ca. 80 % of grade II-III gliomas and in 12 % of GBM patients in total [34,35] and result in overall decrease of NADH-associated fluorescence as non-fluorescent 2-hydroxyglutarate (2-HG) is produced instead of NAD(P)H [36,37]. Similar trends of reduced FAD fluorescence are also observed in tumour when compared with healthy tissue [38]. Both of these bio-markers, as well as PpIX, have been researched extensively as potential identifiers of tumour cells for optical delineation [39]. Previous fluorescence spectroscopy and life-time investigations showed that a superior accuracy of tumour identification can be achieved when a number of fluorescent bio-markers are measured [28,29]. Furthermore, other fluorescent bio-markers such as tryptophan [40,41] and collagen [42,43], for example, may also be critical for tumour cell identification. Hence, an instrument capable of discriminating between multiple native (FAD, HADH) and exogenous (5-ALA/PpIX) fluorescent bio-markers in complex scattering media has a strong potential for clinical translation especially in geographic areas where hospital funding does not allow for state of the art FGR microscopes. This study successfully showed that bio-marker fluorescence intensities in 3D concentration matrix had a linear relationship with their concentration. PLS performed on the data showed high agreement between predicted and actual fluorophore concentration values and maintained the linear relationship. Such capability allows this type of system to be applied to a broader range of applications where tumour discrimination may rely on combination of different fluorophores.

## 5. Conclusion

The work presented here characterised an embedded, miniaturised multi-spectral instrument and compared its performance with a commercial spectrometer. The miniaturisation of optical elements proved to be a viable strategy for spatial separation of diffuse reflectance, fluorescence signals and simultaneously accounting for ambient illumination. The PLS performed on the data collected with the embedded system confirmed the capability of this method to discriminate between three separate fluorophores mixed into liquid tissue phantom. This result, combined with the miniaturised optics shows the strong potential of this technology for integration into existing surgical instruments and significantly improve surgical guidance and quantitative differentiation between healthy tissue and tumour margins. Quantitative decision making, guided by miniaturised optics will result in improved post-operative quality of life, decrease tumour recurrence rates, increase life expectancy and make the ontological treatments even more effective.

## Data Availability

The data is openly available at [44].
